# Non-mutational (epigenomic) structural variation in the transcriptome of cancer cells

**DOI:** 10.18632/oncotarget.3406

**Published:** 2015-01-06

**Authors:** E. Aubrey Thompson

**Affiliations:** Professor of Cancer Biology, Mayo Clinic Florida, Jacksonville, FL, USA

Structural variation at the level of genomic DNA is well-understood to be an important mechanism in malignant transformation. The ability to sequence the whole exome of both normal and malignant cells has recently made it possible to describe all of the single nucleotide and small insertion/deletion mutations within the genome, and total RNA sequencing has made it possible to quantify the abundance of mutated transcripts. Analysis of large structural variants (rearrangements) has lagged somewhat behind the analysis of small sequence variants, but comparative studies of large datasets [1] strongly suggests that some tumor types may be driven by such mutations to a greater or lesser degree.

The role of epigenomic (i.e., non-mutational) changes in transcript sequence is only beginning to be studied in detail. Nevertheless, it is widely believed that programmed changes in promoter utilization, exon inclusion/exclusion, and 3′ end formation are likely to be significant driver processes in transformation. Several examples have been studied in some detail. Notably, the RAC1b splice variant results from an exon inclusion mechanism and results in expression of an oncogenic, constitutively active form of this key regulator of MAPK signaling and actin cytoskeleton dynamics [2].

The recent paper by Bria et al. [3] focuses upon alternative splicing of the ENAH transcript, encoded by the human homolog of the Drosophyla Ena (enabled) and the mouse Mena (murine enabled) gene and called hMENA by the authors. A member of the ena/vasp family of actin regulatory proteins, the primary ENAH transcript is processed to yield several isoforms, including hMENA, hMENA∆6, and hMENA11a, as shown in Table 1.

**Table 1:** 

Chr	start	end	ENAH exon#	hMENA	hMENA-11a	hMENAΔ6
chr1	225674534	225685514	14			
chr1	225686049	225686106	13			
chr1	225688694	225688772	12			
chr1	225692693	225692755	11a			
chr1	225695653	225695719	11			
chr1	225699513	225699561	10			
chr1	225700359	225700416	9			
chr1	225700573	225700718	8			
chr1	225702298	225702602	7			
chr1	225704898	225705008	6			
chr1	225706900	225707267	5			
chr1	225718256	225718340	4			
chr1	225742608	225742785	3			
chr1	225754951	225755116	2			
chr1	225840388	225840845	1			

Splice variants of the ENAH primary transcript can be distinguished by inclusion of exon 11a (hMENA11a) or exclusion of exon 6 (hMENAΔ6).

The translation products of these two alternatively spliced transcripts have been shown to have opposing effects on cellular motility and invasion: hMENA∆6 appears to promote invasion, whereas hMENA11a appears to suppress the invasive phenotype [4]. Alternative splicing of hMENA has been reported to be controlled by the ESRP1/2 splicing factors [5], and the relative abundance of the two hMENA isoforms may play a role in determining the metastatic potential of tumor cells [6].

The current report extends previous observations to include a potential prognostic role for hMENA splice variants in early stage non-small cell lung cancer (NSCLC). An initial analysis of 248 node negative NSCLC patients identified a subset of patients whose tumors did not express detectable levels of hMENA protein. Moreover, survival data indicated that patients with low ratios of total hMENA to hMENA11a had somewhat better outcome. A multifactorial model that incorporated total hMENA/hMENA11a, tumor size, and nodal status was subsequently evaluated in an independent cohort of 133 NSCLC samples. Unfortunately, the validation cohort lacked statistical power to evaluate low risk patients (T1, <10 resected nodes, low ratio total hMENA/hMENA11a). In a comparison of intermediate to high risk patients, the model trended towards discrimination between these two cohorts in disease free and cancer specific survival, but was not significant in a comparison of overall survival between the two groups.

Although the prognostic model remains to be rigorously validated in an appropriately powered cohort of early stage NSCLC, the trend is suggestive and certainly warrants further analysis. It will be even more difficult to assess the predictive power of the model (i.e., the ability to guide therapeutic decision making in the clinic), but it is interesting to speculate on the possible association between hMENA splice variants, actin/cytoskeleton dynamics, and the development of novel therapeutic strategies that directly or indirectly target the actin cytoskeleton.

From a biological standpoint, these studies emphasize the concept that alternative splicing is likely to play a significant role in the cellular phenotype of transformed cells. A more thorough understanding of these epigenomic alterations in transcript structure (and function of the corresponding translation products) is likely to lead to new insight into the process of malignant transformation, and may result in the development of powerful new predictive or prognostic models as well as novel targeted therapies. Although this concept is generally accepted among tumor biologists, and clearly demonstrated in this report, the tools to begin to look at global splicing patterns are, in general, immature. It remains to be seen how this technical issue will be resolved. A great deal remains to be learned about the links between the malignant phenotype and alternative promoter utilization (leading to altered 5′ UTRs), exon inclusion/exclusion (as with hMENA), and 3′ end formation (leading to alternative translational regulatory mechansims). It is nevertheless clear that a fuller understanding of both biological and clinical properties of tumor cells will require a more detailed analysis of oncogenic epigenomic alterations in the nucleotide sequence of mature transcripts.
